# Equine veterinarians' care priorities regarding vaccination, colic, lameness and pre‐purchase scenarios

**DOI:** 10.1111/evj.14537

**Published:** 2025-06-01

**Authors:** Yteke Elte, Inga Wolframm, Hans Vernooij, Mirjam Nielen, René van Weeren

**Affiliations:** ^1^ Faculty of Veterinary Medicine, Department of Clinical Sciences Utrecht University Utrecht The Netherlands; ^2^ Van Hall Larenstein University of Applied Sciences Velp The Netherlands; ^3^ Faculty of Veterinary Medicine, Department of Population Health Sciences Utrecht University Utrecht The Netherlands

**Keywords:** client satisfaction, communication, horse, practice management, quality of care

## Abstract

**Background:**

Equine veterinarians play a crucial role in maintaining equine health and ensuring client satisfaction. Understanding their perspectives on key aspects of veterinary care is essential for optimising outcomes for both clients and horses.

**Objectives:**

To identify and compare the importance equine veterinarians place on seven key aspects of client satisfaction in equine veterinary practice (quality of care, quality of service, horsemanship, interpersonal skills, transfer of knowledge, financial aspects and professionalism) across four different scenarios.

**Study Design:**

A cross‐sectional survey‐based study.

**Methods:**

A total of 246 equine veterinarians participated in the online survey, which included ranking the seven aspects of equine veterinary care across four scenarios. The data were analysed using Friedman tests to assess differences within and across scenarios, followed by Wilcoxon signed‐rank tests with Bonferroni correction for post‐hoc comparisons. Fisher's exact test and Kruskal–Wallis tests were used to determine differences between groups of veterinarians.

**Results:**

Quality of care was ranked most important in the colic (median rank: 1, interquartile range [IQR]: 1–2) and lameness (median rank: 1, IQR: 1–2) scenarios (*p* < 0.001), reflecting the critical nature of these conditions. Quality of service showed no differences in ranking across the scenarios. Professionalism was ranked significantly more important in the pre‐purchase scenario (median rank: 2, IQR: 1–3) compared to other scenarios (*p* < 0.001). Financial aspects were consistently ranked least important (median rank: 7, IQR: 6–7, *p* < 0.001). No differences in ranking were found between different groups of veterinarians.

**Main Limitations:**

Participants may not accurately represent the diversity and characteristics of the entire equine veterinary population. The scenarios do not fully encompass the diversity of equine veterinary practice.

**Conclusions:**

Equine veterinarians prioritise quality of care, quality of service and professionalism in their practice, with some variations depending on the clinical scenario. Financial aspects were consistently given the lowest priority.

## 1 INTRODUCTION

Equine veterinary practice can be highly rewarding for veterinarians. However, horse owners often have high expectations regarding veterinary care for their horses which, in some cases, can lead to stress and ethical dilemmas for all involved.^1^, ^2^ As guardians of equine health and welfare, veterinarians play a pivotal role in maintaining equine health and ensuring the satisfaction of discerning horse owners with their services.^1^, ^3^, ^4^ Awareness of the potential differences in perception and expectations between clients and veterinarians may help the latter optimise the effectiveness of the interaction with their clients, which is likely to have a positive effect on the outcome for the client, veterinarians and horses.^5^

Previous research has demonstrated discrepancies in perceptions regarding veterinary care between equine veterinarians and their clients which may arise due to differences in their knowledge and priorities.^6^ Veterinarians tend to rely on evidence‐based practices and clinical indicators to assess horse health and welfare, while horse owners may prioritise behavioural signs and their horses' well‐being based on personal experience and traditional practices.^7^ When bridging this gap between veterinarians and owners through mutual understanding, owners are more likely to follow veterinary recommendations, resulting in improved health outcomes.^3^

To enhance the quality of care and improve client satisfaction, it is important to understand the perspectives of both horse owners and equine veterinarians.^1^, ^5^ Previous research on client satisfaction in equine veterinary practice has identified seven key aspects important to horse owners, which may also be suitable for determining the priorities of equine veterinarians. To provide a comprehensive understanding of the factors that affect equine veterinary services, it is essential to understand the values and priorities of equine veterinarians when providing veterinary care. While previous studies have primarily focused on clients' expectations and preferences, there is a noticeable gap in the literature regarding veterinarians' perspectives.^1^, ^8^

The objective of this study was to explore which aspects equine veterinarians, either working primarily ambulatory, in a referral clinic, or a combination of both, consider important when managing different types of clinical equine veterinary scenarios.

## 2 MATERIALS AND METHODS

### 2.1 Study design

This study used a survey‐based cross‐sectional design. The survey was created using Qualtrics software, version July 2022 (Qualtrics), and published on the website of the Faculty of Veterinary Medicine of Utrecht University in the Netherlands. The survey link was shared on social media platforms (Facebook and LinkedIn) to achieve further distribution through a snowball effect.^9^

### 2.2 Survey design

The participants were requested to provide their consent on the initial page of the survey. It was emphasised that participation was voluntary and anonymous and that no personal information that could be linked to an individual was collected or saved. Although participants could opt out of the study at any point, once their responses were submitted, the anonymity of the data meant that information could not be removed from the data set.

The survey was available in English and Dutch, and consisted of 20 questions. The completion took an average of 11 min. The complete survey can be found in Survey S1.

The first part of the survey contained ten demographic questions. After the informed consent page, the first question was, ‘Are you a veterinarian?’, if the answer was ‘no’, the participant was routed out of the survey and thanked for their interest. The second question asked about the amount of equine work done by the participants. Only participants who reported spending ≥51% of their veterinary work on horses were included in the final analysis.

In the second part of the survey, participants were introduced (with an infographic) to seven aspects of professional equine veterinary care found to be relevant for equine veterinary care: *quality of care*, *quality of service*, *horsemanship*, *interpersonal skills*, *transfer of knowledge*, *financial aspects* and *professionalism*.^1^

Four scenarios involving different equine veterinary service situations were presented to participants in a fixed order. The scenarios were developed by the first author, who is an experienced equine veterinarian and horse owner, in collaboration with the second author, who is a human behaviour expert, sports psychologist and horse owner. The objective of the scenario design was to incorporate four commonly encountered equine veterinary services: preventative measures (vaccination), emergency calls (colic), standard horse care (lameness) and pre‐purchase assessments.^8^, ^10^, ^11^, ^12^

In the survey, the seven aspects of equine veterinary professional conduct were randomly presented to the participants in all scenarios. The participants were asked to rank these aspects in order of importance, with the most important aspect ranked first (1) and the least important aspect ranked last (7).

Nine equine veterinarians were recruited to conduct a trial run of the survey. They were instructed to focus on the clarity of the questions and scenarios, the survey's operation, potential inconsistencies and the time required to complete it. After the pilot phase, the final version of the survey was released online from 31 October 2022 to 6 June 2023.

### 2.3 Data analysis

**FIGURE 1:**
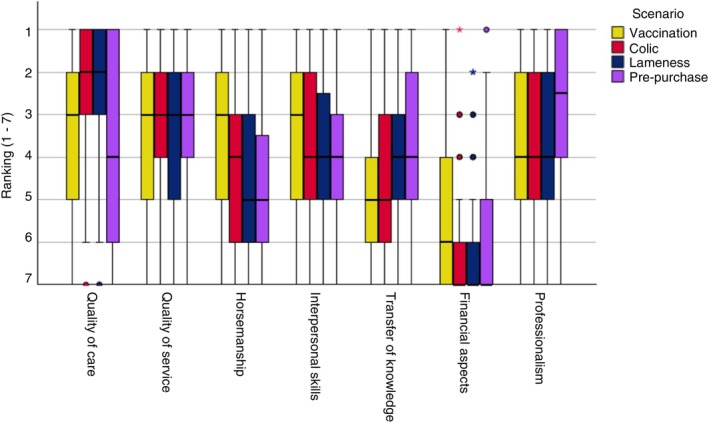
Boxplots representing the ranking distribution of the seven aspects of equine veterinary care for all four scenarios (1 = most important, 7 = least important).

**FIGURE 2:**
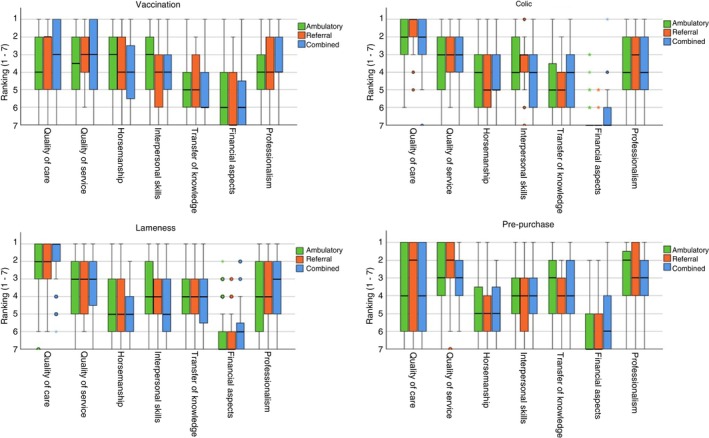
Boxplots representing the ranking distribution of the seven aspects of client satisfaction in equine veterinary practice regarding vaccination, colic, lameness and pre‐purchase per working environment (in an ambulatory, referral, or combination work setting) (1 = most important, 7 = least important).

All surveys with, at a minimum, responses to the questions on the amount of equine work and type of practice in the first part of the survey and with all four scenarios completed, were included in the final statistical analyses. The distribution of the ranking of each aspect within a scenario is depicted in boxplots (Figures 1 and 2).

Non‐parametric tests were used due to the ordinal nature of the data. Friedman tests were conducted to assess differences in ranking across and within the four scenarios for each aspect. When the Friedman test indicated statistical significance, post hoc pairwise comparisons were conducted using Wilcoxon signed‐rank tests and following visual inspection of the boxplots. To control for multiple comparisons, a Bonferroni correction was applied, adjusting the significance threshold to *α* = 0.0125 (0.05/4) for scenario comparisons (four scenarios) and *α* = 0.0071 (0.05/7) for aspect comparisons (seven aspects).

For the pre‐purchase scenario, a comparison was made between respondents stating pre‐purchase as one of the aspects of equine veterinary care they spent the most time on and respondents who did not (Table S1). Fisher's exact test was performed for count data. Kruskal–Wallis tests were conducted to assess differences in the ranking of the seven aspects of veterinary service across different work environments (ambulatory, referral and combined) to determine whether the distribution of rankings differed significantly among these groups for each scenario (Table S2).

The statistical program SPSS version 29.0 was used for the analysis and visualisation.

## 3 RESULTS

The study attracted 291 participants. One participant selected ‘I do not want to participate’ and was removed from the study. After removing entries that answered that they were not veterinarians and/or working with horses 50% or less of their time, 246 responses were included in the final analysis.

All participants who revealed their country (*N* = 173, 71.3%) resided mainly in a country with a well‐developed horse industry (Table 1). Most participants were female, reported doing only equine work, were in paid employment and worked mostly ambulatory in general practice. The detailed demographics of the participants are shown in Table 2. Of the 246 included participants, 205 participated in the vaccination scenario, 200 in the colic scenario, 189 in the lameness scenario and 184 in the pre‐purchase scenario.

**TABLE 1:** Country of residence of the participants.

Country	*N*	%
Australia	7	2.8
Austria	3	1.2
Belgium	4	1.7
Canada	2	0.9
Denmark	1	0.4
Finland	1	0.4
France	2	0.9
Germany	53	21.5
Ireland	5	2.0
Italy	1	0.4
The Netherlands	58	23.6
New Zealand	2	0.9
Romania	2	0.9
Sweden	1	0.4
United Kingdom of Great Britain and Northern Ireland	21	8.5
United States of America	10	4.0
Unknown	73	29.7
Total	246	100

**TABLE 2:** Participant demographic information.

Demographic data		*N*	%
Equine work	51%–99%	53	21.5
	100%	193	78.5
Employment status	Self‐employed/solo practitioner	44	17.9
	Self‐employed, with support staff	20	8.1
	Practice owner/partnership member	39	15.9
	Paid employment	128	52.0
Type of practice	>80% Ambulatory work	142	57.8
	>80% Referral clinic	39	15.9
	Combination ambulatory‐referral	50	20.3
	Other/no choice	15	6.1
Type of work^a^	General practice	198	78.2
	Orthopaedics	74	29.2
	Internal medicine	78	30.8
	Sport horse medicine	60	23.4
	Reproduction	80	31.6
	Pre‐purchase examinations	48	19.0
	Dentistry	84	33.2
	Surgery	21	8.3
Gender	Male	56	22.8
	Female	173	70.3
	Non‐binary	1	0.4
	Rather not say	1	0.4
	Other/no choice	15	6.1
Age	18–24	1	0.4
	25–34	84	34.1
	35–44	74	30.1
	45–54	43	17.5
	55–64	23	9.3
	65–74	5	2.0
	75–84	1	0.4
	No choice	15	6.1

^a^ Multiple answers were possible.

Friedman tests revealed significant differences (*α* = 0.0125) across the scenarios for six of the seven aspects:

*Quality of care*: *χ* ^2^ (3) = 95.2, *p* < 0.001.

*Quality of service*: *χ* ^2^ (3) = 8.84, *p* = 0.032.

*Horsemanship*: *χ* ^2^ (3) = 74.40, *p* < 0.001.

*Interpersonal skills*: *χ* ^2^ (3) = 14.98, *p* = 0.002.

*Transfer of knowledge*: *χ* ^2^ (3) = 43.20, *p* < 0.001.

*Financial aspects*: *χ* ^2^ (3) = 60.82, *p* < 0.001.

*Professionalism*: *χ* ^2^ (3) = 49.50, *p* < 0.001.

Quality of care: Post‐hoc Wilcoxon tests demonstrated that *quality of care* was ranked significantly more important in the colic (*Z* = −7.328, *p* < 0.001) and lameness (*Z* = −6.716, *p* < 0.001) scenarios than in the vaccination scenario. *Quality of care* was also ranked significantly more important in the colic than in the pre‐purchase scenario (*Z* = −7.779, *p* < 0.001) (Figure 1).

Quality of service: No significant differences in the ranking of *quality of service* were observed across the four scenarios (Figure 1).

Horsemanship: *Horsemanship* was ranked significantly more important in the vaccination scenario than in the colic (median 4) (*Z* = −4.735, *p* < 0.001), lameness (*Z* = −6.740, *p* < 0.001), and pre‐purchase scenarios (*Z* = −6.429, *p* < 0.001) (Figure 1). Differences in ranking between the lameness and pre‐purchase scenarios were also significant (*Z* = −3.725, *p* < 0.001) (Figure 1).

Interpersonal skills: *Interpersonal skills* were ranked significantly more important in the vaccination scenario (median 3) than in the lameness (*Z* = −2.727, *p* = 0.006) and pre‐purchase scenario (*Z* = −3.298, *p* = 0.001) (Figure 1).

Transfer of knowledge: *Transfer of knowledge* was ranked significantly less important in the vaccination scenarios than in the lameness (median 4) (*Z* = −4.212, *p* < 0.001) and pre‐purchase scenarios (*Z* = −5.536, *p* < 0.001) (Figure 1).

Financial aspects: *Financial aspects* were ranked least important across all four scenarios (Figure 1). Wilcoxon signed‐rank tests showed significant differences between the vaccination and colic scenarios (*Z* = −6.450, *p* < 0.001) and colic and lameness scenarios (*Z* = −4.393, *p* < 0.001).

Professionalism: *Professionalism* was ranked significantly more important (median 2.5) in the pre‐purchase scenario compared to the vaccination (*Z* = −4.633, *p* < 0.001) and colic (*Z* = −5.314, *p* < 0.001) scenarios (median 4) (Figure 1).

Overall comparison across aspects, within scenarios: A Friedman test comparing the seven aspects of veterinary service within the vaccination scenario was statistically significant (*α* = 0.0071), *χ* ^2^ (6) = 168.300, *p* < 0.001. Wilcoxon post hoc tests showed significant differences between *financial aspects* and all other aspects (*Z* = −7.075 to −8.827, *p* < 0.001) were observed.

Analysis of ranking between aspects within the colic scenario was also significant, Friedman: *χ* ^2^ (6) = 419.751, *p* < 0.001. *Financial aspects* were ranked significantly less important than all other aspects (*Z* = −9.750 to −11.706, *p* < 0.001). Significant differences were also found between *quality of care* and *horsemanship* (*Z* = −8.907, *p* < 0.001), *interpersonal skills* (*Z* = −6.887, *p* < 0.001) and *transfer of knowledge* (*Z* = −9.955, *p* < 0.001).

For the lameness scenario, the Friedman test was significant with *χ* ^2^ (6) = 349.833, *p* < 0.001. Significant differences were found between *financial aspects* and all other aspects (*Z* = −7.827 to −11.648, *p* < 0.001) and between *quality of care* and *horsemanship* (*Z* = −9.425, *p* < 0.001), *interpersonal skills* (*Z* = −7.604, *p* < 0.001) and *transfer of knowledge* (*Z* = −8.463, *p* < 0.001).

For the pre‐purchase scenario, the Friedman test was significant, *χ* ^2^ (6) = 244.428, *p* < 0.001. Significant differences were found between *financial aspects* and all other aspects (*Z* = −5.406 to −10.695, *p* < 0.001). Additional significant differences were observed between *horsemanship* and *professionalism* (*Z* = −7.763, *p* < 0.001), *interpersonal skills* and *professionalism* (*Z* = −6.345, *p* < 0.001) and *transfer of knowledge* and *professionalism* (*Z* = −4.719, *p* < 0.001).

Other: No significant differences in ranking were observed in the pre‐purchase examination scenario between the participants who frequently performed pre‐purchase examinations and those who did not (Table S1). Similarly, there were no significant differences in ranking across working environments (ambulatory, referral or combined) (Figure 2 and Table S2).

## 4 DISCUSSION

The objective of this study was to explore which aspects equine veterinarians, working primarily ambulatory, in a referral clinic, or a combination of both, consider most important when attending to different types of common clinical equine veterinary scenarios.

The results demonstrate that equine veterinarians consider quality of care most important, which mirrors findings of previous research.^6^, ^13^, ^14^ A study investigating horse owners' perceptions of equine veterinary care using the same scenarios found that quality of care was the most important for them.^5^ In addition, quality of care was ranked significantly more important in both the colic and lameness scenarios than in the vaccination and pre‐purchase scenarios. The explanation might be that in the colic and lameness scenarios a veterinary (presumptive) diagnosis and treatment favourable to the horse's health is the desired instantaneous outcome, making quality of care a pivotal parameter. Horse health status is less directly affected by vaccination or pre‐purchase examination.

Quality of service was ranked either first or second by the participants in all four scenarios, with no significant differences, complementing findings in earlier work that stressed the importance of service quality from the viewpoint of the (equine) veterinary client.^1^, ^5^, ^13^, ^15^, ^16^ Service quality can be defined as the ability of a service provider to consistently meet or surpass the expectations of their clients, making it pivotally important to client satisfaction levels.^17^ True failure in quality of service, such as responding late to an emergency, can even be an important reason for filing a complaint with a disciplinary board.^15^ The current findings show that veterinarians are aware of the additional value of their services, in addition to their veterinary skills. This is reassuring and presents an opportunity to achieve greater levels of satisfaction among clients by offering superior services.

Veterinarians in this study ranked horsemanship significantly more important in the vaccination scenario (median rank 3) than in the colic (median rank 4), lameness (median rank 5) and pre‐purchase scenarios (median rank 5), and at the same level as the horse owners in an earlier study.^5^ This is possibly because of the animal handling skills involved; vaccination of the needle‐shy horse is a frequent occurrence in equine veterinary practice and a common stressor for all involved, including the horse.^18^, ^19^ In the colic and lameness scenarios, the focus is primarily on diagnosing and treating the underlying condition rather than on how the horse is handled during the procedure. Horsemanship was ranked lowest by veterinarians in the pre‐purchase scenario. In summary, where the way in which veterinarians deal with a client's horse is of great importance, the relative importance of horsemanship probably varies based on the immediacy and complexity of the veterinary intervention required and the type of horse owner involved.^1^, ^5^, ^8^

Interpersonal skills were ranked as moderately important by the participants, with a significantly higher ranking in the vaccination, than in the lameness and pre‐purchase scenario. This might suggest that veterinarians prioritise communication and client interaction more in routine preventive care settings. Blach made a similar observation in a paper in which it was stated that while competence in veterinary skills is paramount, interpersonal skills are still crucial for client satisfaction.^13^ Horse owners ranked interpersonal skills lower in the study by Elte et al. than in the current study.^5^ Mellanby et al. also found that veterinarians often emphasise the importance of good communication skills, whereas clients tend to prioritise technical knowledge and practical skills over interpersonal attributes.^6^ This finding suggests that while interpersonal skills are recognised as important, they may not be seen as a distinguishing factor of a ‘good veterinarian’ by clients compared to more tangible skills and outcomes. However, when clients are dissatisfied, they mention personal demeanours and communication as one of the main reasons for dissatisfaction.^20^ Veterinarians, on the other hand, are likely much more aware of the importance of interpersonal skills, either from experience or through the growing emphasis on interpersonal skills in veterinary curricula.^21^, ^22^ Haldane et al. even stated that ‘Veterinarians and students ranked verbal communication and interpersonal skills as the most important skill set for an entry‐level veterinarian’.^22^ However, there seem to be some differences in the perception of this topic between clients and veterinarians.

Current data indicates that participants ranked transfer of knowledge significantly more important in the lameness and pre‐purchase scenarios (median rank 4) than in the vaccination scenario (median rank 5). Clients did the same, with the highest importance for transfer of knowledge in the pre‐purchase scenario.^5^ Horse owners and veterinary clients, in general, prefer to be educated about the condition of their animals.^14^ It could be argued that veterinary findings related to either lameness or the purchase of a horse can have major implications for future performance; therefore, for the owner, transfer of knowledge could be most relevant in these scenarios. There is a shared recognition of the critical impact that informed decision‐making can have on the long‐term health and performance of horses, which underscores the essential role of effective communication and education in veterinary practice, particularly in scenarios where stakes are high.^5^, ^11^, ^23^, ^24^

Equine veterinarians ranked financial aspects the least important among all four scenarios. This is consistent with the client's perspective, as reported in an earlier study by Elte et al.^5^ This may be interpreted as equine veterinarians and horse owners prioritising the health and welfare of the horse and seeing their profession or their relationship with the horse as primarily driven by a commitment to animal care rather than financial motivation.^1^, ^5^ Studies suggest that horse owners are willing to invest in the necessary treatments and interventions to ensure their horses' health, regardless of the cost.^8^, ^23^ However, Burrell et al. identified a different approach to the financial aspects of veterinary care.^25^ In that study, horse owners reported financial aspects as a concern, although not the most important in their decision‐making process, and the veterinarians saw costs as the largest influencer in decision‐making and felt pressured to secure payment.^25^ A difference between that study and ours is that the study by Burrell et al. was a retrospective study about decisions taken on a limited number (14) of cases of severe colic, and our study was based on a survey in a larger population asking what people believe is most important in given cases. In this regard, social desirability bias may have influenced our results.^26^ The way individuals self‐present themselves can differ in a research setting and in a naturalistic environment.^27^ Acknowledging financial considerations might be perceived as less noble or altruistic, even though financial constraints can significantly impact decision‐making in veterinary care.^25^, ^28^, ^29^, ^30^ This potential bias emphasises the need for careful interpretation of survey data, recognising that the expressed priorities might not fully capture the complex interplay between financial and ethical considerations in equine veterinary care.^31^

In general, veterinarians (and horse owners) prioritise care quality and outcomes over financial considerations. However, cost‐effectiveness, the way in which financial aspects are addressed, and the pressure experienced by veterinarians are important issues in equine veterinary care provision.^13^, ^25^, ^32^

Veterinary professionalism is marked by veterinarians' conduct with clients, encompassing accuracy, attire, cleanliness and virtues, such as honesty, trustworthiness and respect.^1^, ^33^ Equine veterinarians ranked professionalism significantly more important for pre‐purchase examinations than for the vaccination and colic scenarios. First, pre‐purchase examinations often involve significant financial investments for the buyer and potential legal implications for all involved (including the veterinarian), necessitating a high level of professionalism to ensure thorough and accurate evaluations of the horse's health and suitability for purchase. Second, pre‐purchase examinations typically require detailed documentation and correct communication with all involved parties, including the buyer, seller and other representatives, highlighting the importance of professionalism in managing expectations and facilitating transparent transactions.^11^ Further, the complex nature of pre‐purchase examinations, which often involve comprehensive physical examinations, diagnostic imaging and extensive discussion of findings, demands professional skills to navigate potential conflicts of interest and uphold the integrity of the veterinary profession. Interestingly, the current study shows no significant difference in the ranking of the aspects between veterinarians who regularly perform pre‐purchase examinations and those who do not, which means that awareness of the need for a strict professional attitude during pre‐purchase exams is deeply rooted in the profession.

The current study found no differences in the ranking of the seven aspects across all scenarios, irrespective of whether the veterinarians worked in ambulatory, referral, or combination settings. Interestingly, this uniform perception contrasts with existing research, indicating notable differences in care perception based on setting.^34^, ^35^, ^36^ According to May (2015), there are distinct differences between primary and referral care settings in terms of operation and expertise.^36^ Primary care focuses on a broad, holistic approach, addressing common and less defined conditions with a focus on resolving the issue, while referral care deals with specific, often severe, cases requiring specialised diagnostic and therapeutic procedures, with more focus on the final diagnosis.^36^ It could be that the perceived difference lies not in the aspect itself but in its presentation across settings. Study setup might also influence outcomes, as perceptions may vary when specifically asked about a referral setting compared to ranking the importance of an aspect within a scenario.^27^

## 5 LIMITATIONS

The participants in the study may not accurately represent the diversity and characteristics of the entire equine veterinary population. For example, participants who spend less than 50% of their time working with horses could have a different perspective. Participants were asked twice about their identity as equine veterinarians; however, the correctness of their answers was not verified. These scenarios do not fully encompass the diverse range of activities and challenges encountered in equine veterinary practice.

## 6 CONCLUSION

This study explored equine veterinarians' prioritisation of seven aspects of veterinary care known to be key elements in client satisfaction in different clinical scenarios. Reflecting their dedication to achieving optimal health outcomes for horses, vets rank quality of care and service highest, as do their clients. Professionalism scores particularly high during pre‐purchase examinations. Financial aspects are consistently ranked lowest by veterinarians, paralleling horse owners' views, suggesting a mutual prioritisation of horse welfare over cost, and likely an ethical commitment to animal care. However, in this aspect, there may be social desirability bias, with financial considerations (not unlikely) underreported to conform to social norms and professional ideals. Our study did not find any differences in the ranking of the seven aspects among veterinarians in different settings (ambulatory, referral, or combined). The knowledge that there is very good agreement with the prioritisation of these aspects with horse owners provides a good basis for the optimisation of equine veterinary care and the shared decision‐making process, which in itself provides an opportunity to improve both client satisfaction and job satisfaction of the vet, ultimately enhancing horse health.

## FUNDING INFORMATION

Not applicable.

## CONFLICT OF INTEREST STATEMENT

The authors declare that they have no conflicts of interest.

## AUTHOR CONTRIBUTIONS

**Yteke Elte:** Conceptualization; investigation; writing – original draft; methodology; data curation; writing – review and editing; validation. **Inga Wolframm:** Conceptualization; investigation; writing – original draft; methodology; supervision; validation; writing – review and editing. **Hans Vernooij:** Writing – review and editing; formal analysis; visualization. **Mirjam Nielen:** Supervision; writing – review and editing; conceptualization. **René van Weeren:** Conceptualization; writing – review and editing; supervision.

## DATA INTEGRITY STATEMENT

Y. Elte had full access to all the data in the study and takes responsibility for the integrity of the data and the accuracy of the data analysis. The data that support the findings of this study are openly available in DataverseNL, https://doi.org/10.34894/RKAZCE.

## ETHICAL ANIMAL RESEARCH

The study was approved by the Science‐Geosciences Ethics Review Board of Utrecht University in the Netherlands (approval number: DGK S‐22744).

## INFORMED CONSENT

Participants gave consent for inclusion in the study.

## Supporting information


**Survey S1.** Survey on the expectations of veterinarians regarding equine veterinary services.

**Table S1.** Distribution of ranking of the seven aspects of client satisfaction in equine veterinary practice for the pre‐purchase scenario, showing differences between veterinarians who regular perform pre‐purchase examinations and those that do not.

**Table S2.** Kruskal–Wallis test results comparing the ranking of the seven aspects across different work environments (ambulatory, referral clinic and combined practice) within each scenario (vaccination, colic, lameness and pre‐purchase).

## Data Availability

The data that support the findings of this study are openly available in DataverseNL, https://doi.org/10.34894/RKAZCE.
