# Supplementary material for: Equine veterinarians' care priorities regarding vaccination, colic, lameness and pre‐purchase scenarios
Source: Equine Vet J. 2025 Jun 1;58(1):203–11. doi: 10.1111/evj.14537 (PMC12699120; doi:10.1111/evj.14537)
Supplement: Supplementary file 1 — Survey S1. Survey on the expectations of veterinarians regarding equine veterinary services. [file EVJ-58-203-s003.pdf]

Equine Veterinary Journal BEVA

**Survey S1:**

# Vet assumptions regarding client expectations

Start of Block: Default Question Block

Q1
Survey on the expectations of veterinarians regarding equine veterinary services

INFORMATION AND CONSENT

You are invited to participate in a survey into the expectations and actions of horse owners and keepers regarding veterinary care for horse(s). This research is being conducted by Drs. Yteke Elte, veterinarian and researcher at the Faculty of Veterinary Medicine at Utrecht University.

What is expected of you?

Participating in the survey means that you will complete an online questionnaire. The questions relate to you as an equine veterinarian and what you consider important when providing veterinary service. Filling in the questionnaire takes approximately 10 minutes.

Voluntary

You voluntarily participate in this research. Therefore, you may end your participation and withdraw your consent at any time during the study. You do not have to indicate why you no longer wish to participate. Because the data is immediately anonymized, it is not possible to have your research data deleted after the questionnaire has been completed.

What happens with my data?

The research data we collect in this study will be used by the researchers for data analysis, articles and presentations. The anonymized research data will be available to other researchers for at least 10 years. If we share data with other researchers, it cannot be traced back to you. We store all research data in a secure manner in accordance with the guidelines of Utrecht University.

Ethical review, questions and complaints

This study has been approved by the Ethical Review Committee DGK S-22744
Do you have any questions, worries or a complaint about your privacy? Please contact: privacy@uu.nl

Do you have questions about the research?
Please contact the responsible researcher, Drs. Yteke Elte,
Telephone: +31 30 2531111
Email: y.elte@uu.nl

PERMISSION:  Please indicate your choice below.

By clicking on the 'I agree' button you indicate that you:
● have read the above information
● voluntarily participate in the study
● are 16 years or older

If you do not want to participate in the study, you can click on the 'I do not want to participate' button

○ I agree, continue to the questionnaire  (1)

○ I do not want to participate  (2)

Page Break

Q39 Are you a veterinarian?

◯ Yes (1)

◯ No (2)

*Skip To: End of Survey If Ben je dierenarts? = Nee*

Q6 What percentage of your veterinary work is related to horses?

◯ I do not work with horses (1)

◯ <50% (7)

◯ 51-99% (8)

◯ 100% (9)

*Skip To: End of Survey If Hoeveel procent van je werk besteed je aan de paardengeneeskunde? = Ik behandel geen paarden*

X→

country In which country do you currently reside?

▼ Afghanistan (1) ... Zimbabwe (1357)

Q2 Are you?

- ○ Male (1)
- ○ Female (2)
- ○ Non- binary (3)
- ○ Other (4)
- ○ Rather not say (5)

---

Q3 How old are you?

▼ 16 (1) ... 90 (82)

---

Q40 How many years have you been working as an equine veterinarian?

▼ 1 (1) ... 50 (50)

---

Q7 Which aspects of equine veterinary care do you spend most of your time on? Choose 3 max.

- ☐ General practice (1)
- ☐ Orthopedics (2)
- ☐ Internal medicine (3)
- ☐ Sport horse medicine (4)
- ☐ Reproduction (5)
- ☐ Pre purchase examinations (6)
- ☐ Dentistry (7)
- ☐ Surgery (17)

Q8 Are you a board-certified specialist with either an European or American college? (e.g. ECEIM, ECVS, ACVR, ACVSMR, etc.)

- ○ Yes (1)
- ○ No (2)

Q42 Which description best fits your work situation? Choose 1.

○ I am self-employed/ solo practitioner (1)

○ I am self-employed, but I do have support staff (2)

○ I am a practice owner/partnership member. How many vets work in your practice? (5) ________________________________________________

○ I work in paid employment. How many vets work in your practice? (6) ________________________________________________

Q47 Which description best fits your work environment? Choose 1.

○ I mainly do ambulatory work (1)

○ I mainly work in a (referral) clinic (2)

○ I work both in an ambulatory setting and in a (referral) clinic (3)

**End of Block: Default Question Block**

**Start of Block: Block 1**

Q15 A healthy and happy horse, that's what most horse owners and care takers aim for. To achieve this, most horses need (preventive) veterinary care at some point in their lives. Research has shown that there are 7 aspects that are important to horse owners when it comes to veterinary services. You can find these in the infographic below.

We would like to know what you, as an equine veterinarian, believe is most important when it comes to providing veterinary care and the interaction with horse owners or care takers.

We've written **four scenarios** that may seem familiar to you. Please indicate for each scenario which of the 7 aspects of veterinary services are most important to you by rating them in order of preference.

Q34

Q34

Scenario 1 **Scenario 1**

It's time for the annual influenza vaccination.
The owner schedules an appointment with you.

Which aspect do you, as a vet, consider most import in this scenario?

Below you will find the aforementioned seven aspects of the veterinary care provided. See the infographic for more information.

Put the item you believe is most important to you at number 1 and the least important at number 7.

______ Quality of care (1)
______ Quality of service (2)
______ Horsemanship of the veterinarian (3)
______ Interpersonal skills (4)
______ Transfer of knowledge (5)
______ Cost of service (6)
______ Professionalism (7)

End of Block: Block 1

Start of Block: Block 2

Page Break

Q35

Q35

Q19
**Scenario 2**

It's early evening, the owner receives a phone call telling him/her that there is something the matter with his/her horse. The owner panics and rushes to the stable straight away. By the time s/he gets there, the horse has got himself cast in his stable. He is soaked in sweat, and covered in straw with a scrape above his eye. The bed looks completely trashed. The owner enlists the help of a few people at the yard and together, they manage to get the horse to his feet. The horse paws at the ground and immediately wants to lie down again. The afternoon feed lies untouched in the manger. The owner manages to get the horse to the arena. Once there the horse immediately lies down again.
The owner calls you to check the horse.
Which aspect do you, as a vet, consider most import in this scenario?

Put the item you believe is most important to you at number 1 and the least important at number 7.
______ Quality of care (1)
______ Quality of service (2)
______ Horsemanship of the veterinarian (3)
______ Interpersonal skills (4)
______ Transfer of knowledge (5)
______ Cost of service (6)
______ Professionalism (7)

End of Block: Block 2

Start of Block: Block 3

Page Break

Q37

Q37

Q31 It is a beautiful late summer evening. It is already getting dark, but the horses are still in the field. One of the owners is busy in the yard. Suddenly he/she sees that the horses are all running across the field, heading straight for the fence. Most of them manage to stop in time. Only this owner's horse jumps a little clumsily over the fence. On landing, he slips on the dirt track leading to the fields. He gets up straight away again and trots into the yard. At first glance, he does not appear to be lame and the owner can't see any injuries. He/she puts the horse in the stable. The next day though, the horse seems to be a bit stiff which, even after a few days, doesn't get any better.

The owner calls you to check the horse.

Which aspect do you, as a vet, consider most import in this scenario?

Put the item you believe is most important to you at number 1 and the least important at number 7.
______ Quality of care (1)
______ Quality of service (2)
______ Horsemanship of the veterinarian (3)
______ Interpersonal skills (4)
______ Transfer of knowledge (5)
______ Cost of service (6)
______ Professionalism (7)

**End of Block: Block 3**

**Start of Block: Block 4**

Page Break

Q39

Q39

---

Q29

An owner has been scouring adverts and travelling through the country for weeks, in search of a new horse (for him/herself or for a client). The search hasn't been in vain. He/she has found a great horse and now wants to buy her.

The owner schedules an appointment with you for a pre purchase examination.

Which aspect do you, as a vet, consider most import in this scenario?

Put the item you believe is most important to you at number 1 and the least important at number 7.

______ Quality of care (1)
______ Quality of service (2)
______ Horsemanship of the veterinarian (3)
______ Interpersonal skills (4)
______ Transfer of knowledge (5)
______ Cost of service (6)
______ Professionalism (7)

---

Q45 Anything else you would like to comment on or share your thoughts on expectations regarding equine veterinary services? You can do this here. (optional)

________________________________________________________________

________________________________________________________________

________________________________________________________________

________________________________________________________________

**End of Block: Block 4**
