# Supplementary material for: Equine veterinarians' care priorities regarding vaccination, colic, lameness and pre‐purchase scenarios
Source: Equine Vet J. 2025 Jun 1;58(1):203–11. doi: 10.1111/evj.14537 (PMC12699120; doi:10.1111/evj.14537)
Supplement: Supplementary file 2 — Table S1. Distribution of ranking of the seven aspects of client satisfaction in equine veterinary practice for the pre‐purchase scenario, showing differences between veterinarians who regular perform pre‐purchase examinations and those that do not. [file EVJ-58-203-s002.pdf]

Equine Veterinary Journal BEVA

Table S1: Distribution of ranking of the seven aspects of client satisfaction in equine veterinary practice for the pre-purchase scenario, showing differences between veterinarians who regular perform pre-purchase examinations and those that do not (1= most important, 7= least important).

		ranking 1	ranking 2	ranking 3	ranking 4	ranking 5	ranking 6	ranking 7
Quality of care (p=0.093)	PPE-vet	6	5	3	6	6	6	9
	Non PPE-vet	49	13	7	11	21	27	15
Quality of service (p=0.26)	PPE-vet	14	8	5	4	7	2	1
	Non PPE-vet	31	34	34	13	13	15	3
Horsemanship (p=0.045)	PPE-vet	5	6	4	6	5	9	6
	Non PPE-vet	2	12	17	21	38	35	18
Interpersonal skllls (p=0.44)	PPE-vet	6	3	10	9	5	7	1
	Non PPE-vet	9	13	21	39	25	27	9
Transfer of knowledge (p=0.98)	PPE-vet	3	6	11	7	6	6	2
	Non PPE-vet	10	28	32	21	24	18	10
Financial aspects (p=0.11)	PPE-vet	0	3	0	2	6	9	21
	Non PPE-vet	2	8	10	16	11	14	82
Professionalism (p=0.71)	PPE-vet	7	10	8	7	6	2	1
	Non PPE-vet	40	35	22	22	11	7	6
Pre-purchase examination (PPE)								
Non PPE-vet n=143								
PPE-vet n=41								
