# Supplementary material for: Equine veterinarians' care priorities regarding vaccination, colic, lameness and pre‐purchase scenarios
Source: Equine Vet J. 2025 Jun 1;58(1):203–11. doi: 10.1111/evj.14537 (PMC12699120; doi:10.1111/evj.14537)
Supplement: Supplementary file 3 — Table S2. Kruskal–Wallis test results comparing the ranking of the seven aspects across different work environments (ambulatory, referral clinic and combined practice) within each scenario (vaccination, colic, lameness and pre‐purchase). [file EVJ-58-203-s001.pdf]

Table S2: Kruskal-Wallis test results (mean rank values) comparing the ranking of the seven aspects across different work environments (ambulatory, referral clinic, and combined practice) within each scenario (vaccination, colic, lameness, and pre-purchase).

**Vaccination**	**Ambulatory (>80%) (N=116)**	**Referral (>80%) (N=29)**	**Combined (N=39)**	**Kruskal-Wallis H**	**df**	**p-value**
Quality of care	96.43	84.19	86.99	1.802	2	0.406
Quality of service	95.70	89.05	85.55	1.239	2	0.538
Horsemanship	87.06	96.28	105.86	3.906	2	0.142
Interpersonal skills	87.67	102.71	99.28	2.725	2	0.256
Transfer of knowledge	92.03	89.84	95.87	0.245	2	0.885
Financial aspects	91.75	95.69	92.36	0.144	2	0.930
Professionalism	96.57	91.53	81.12	2.543	2	0.280
**Colic**	**Ambulatory (>80%) (N=116)**	**Referral (>80%) (N=29)**	**Combined (N=39)**	**Kruskal-Wallis H**	**df**	**p-value**
Quality of care	94.33	79.93	96.41	2.178	2	0.337
Quality of service	92.80	92.64	91.50	0.018	2	0.991
Horsemanship	89.31	101.78	95.09	1.432	2	0.489
Interpersonal skills	89.92	87.95	103.56	2.226	2	0.329
Transfer of knowledge	93.22	100.02	84.76	1.480	2	0.477
Financial aspects	95.45	94.21	82.46	3.027	2	0.220
Professionalism	94.50	88.84	89.28	0.454	2	0.797
**Lameness**	**Ambulatory (>80%) (N=116)**	**Referral (>80%) (N=29)**	**Combined (N=39)**	**Kruskal-Wallis H**	**df**	**p-value**
Quality of care	95.26	91.36	85.14	1.205	2	0.547
Quality of service	90.02	92.26	100.05	1.071	2	0.586
Horsemanship	90.11	100.19	93.90	0.890	2	0.641
Interpersonal skills	88.00	92.16	106.13	3.472	2	0.176
Transfer of knowledge	94.85	87.28	89.40	0.656	2	0.720
Financial aspects	95.04	92.50	84.95	1.287	2	0.526
Professionalism	95.62	87.88	86.67	1.109	2	0.574
**Pre-purchase**	**Ambulatory (>80%) (N=116)**	**Referral (>80%) (N=29)**	**Combined (N=39)**	**Kruskal-Wallis H**	**df**	**p-value**
Quality of care	92.73	87.84	95.28	0.344	2	0.842
Quality of service	92.90	86.52	95.77	0.540	2	0.763
Horsemanship	90.41	85.55	103.87	2.536	2	0.281
Interpersonal skills	90.93	95.88	94.65	0.291	2	0.864
Transfer of knowledge	90.79	103.88	89.12	1.650	2	0.438
Financial aspects	95.39	93.52	83.15	1.892	2	0.388
Professionalism	91.87	96.07	91.72	0.161	2	0.922
